# Supplementary material for: SyNDI: synchronous network data integration framework
Source: BMC Bioinformatics. 2018 Nov 6;19:403. doi: 10.1186/s12859-018-2426-5 (PMC6219086; doi:10.1186/s12859-018-2426-5)
Supplement: Supplementary file 5 — Files of the example presented in Additional file 4. This additional file is a zip package that contains all files used in Additional file 4. Detailed descriptions of each of these files are presented in a README file included in this zip package. (GZ 30411 kb) [file 12859_2018_2426_MOESM5_ESM.gz › SyNDITools/Meme2Fimo/meme_4.9.0_4.tar.gz!/meme_4.9.0/doc/overview.html]

The MEME suite of motif-based sequence analysis tools


The MEME Suite allows the biologist to discover novel motifs in
collections of unaligned DNA or protein sequences and to search
for motif occurrences in sequence databases. The suite is
comprised of a collection of tools that work together, as shown
below. Not all the tools are avaliable as webservices, so to get
the full power of the MEME Suite you will need to
download and install a local
copy of the software.


|  |  |  |  |
| --- | --- | --- | --- |
| **Motif Discovery** | | | |
| MEME | Find ungapped motifs in unaligned DNA, RNA or protein sequences. | Sample output |  |
| MEME ChIP | Automate analysis of ChIP-seq and other large DNA datasets using the MEME suite. | Sample output, |  |
| DREME | Find short motifs in large sets of DNA or RNA sequences. Also allows discriminative motif analysis using a set of control sequences. | Sample output |  |
| Glam2 | Find **gapped** motifs in unaligned DNA or protein sequences. | Sample output, Manual, Tutorial |  |
| **Motif Enrichment Analysis** | | | |
| AME | Motif Enrichment Analysis: find known DNA motifs that are enriched in the input sequences. |  |  |
| CentriMo | Central Motif Enrichment Analysis: Find motifs that are enriched near the centers of equal-length sequences. | Sample output, |  |
| SpaMo | Motif Spacing Analysis: Find known motifs that occur with preferred spacings relative to a primary motif in a set of DNA sequences. | Sample output, |  |
| GOMO | Genome Ontology Motif Enrichment: Identify possible roles (Gene Ontology terms) for DNA binding motifs. |  |  |
| **Motif Search** | | | |
| FIMO | Search a sequence database for occurrences of known motifs. This program treats each motif independently and reports all putative motif occurrences below a specified *p*-value threshold. | Sample output |  |
| MAST | Search a sequence database for occurences of known motifs. This program assumes exactly one occurrence of each motif per sequence, and each sequence in the database is assigned a *p*-value, based on the product of the *p*-values of the individual motif occurrences in that sequence. | Sample output |  |
| MCAST | Search a sequence database for **clusters** of known motifs. `mcast` employs a motif-based hidden Markov model, using a star topology and a novel scoring algorithm. The motifs may appear in any order. | Sample output |  |
| Glam2Scan | Search for occurences of gapped motifs, discovered by GLAM2. | Sample output, Manual |  |
| **Motif Comparison** | | | |
| Tomtom | Find motifs that are similar to a given DNA motif by searching a database of known motifs. | Sample output |  |
| **Additional Primary Tools** | | | |
| AMA | Print the Average Motif Affinity score of each sequence in a database. The score is calculated by averaging the likelihood ratio scores for all feasible binding events to the given sequence and to its reverse strand. |  |  |
| **File Format Conversion Utilities** | | | |
| beeml2meme | Convert an BEEML matrix file to MEME format. |  |  |
| chen2meme | Convert a CHEN matrix file to MEME format. |  |  |
| clustalw2fasta | Convert a Clustalw multiple alignment into FASTA format. |  |  |
| clustalw2phylip | Convert a Clustalw multiple alignment into Phylip format. |  |  |
| glam2format | Convert glam2 motifs to standard alignment formats. |  |  |
| obo2dag | Convert a Gene Ontology OBO file into a GO DAG file. |  |  |
| iupac2meme | Convert an IUPAC string to MEME format. |  |  |
| jaspar2meme | Convert a directory of JASPAR files to MEME format. |  |  |
| mast2txt | Convert MAST XML output into plain text. |  |  |
| meme2meme | Convert and merge multiple MEME formatted files. |  |  |
| priority2meme | Convert a PRIORITY matrix file to MEME format. |  |  |
| readseq | Format conversion utility for sequence data. |  |  |
| rna2meme | Convert a FASTA file with short RNA sequences into motifs for DNA they might bind in MEME format. |  |  |
| scpd2meme | Convert an SCPD matrix file to MEME format. |  |  |
| tamo2meme | Convert a TAMO matrix file to MEME format. |  |  |
| transfac2meme | Convert a TRANSFAC matrix file to MEME format. |  |  |
| uniprobe2meme | Convert a UNIPROBE matrix file to MEME format. |  |  |
| **Other Utilities** | | | |
| alphtype | Classify a string passed as a command line argument as an instance of the DNA or protein alphabet. |  |  |
| ama-qvalues | Add q-values to ama output. |  |  |
| ceqlogo | Create motif logos. |  |  |
| compute-prior-dist | Compute the distribution of priors in a MEME PSP format file. | Sample output |  |
| fasta-get-markov | Estimate a Markov model from a FASTA file of sequences. |  |  |
| fasta-io | Read and write FASTA files. |  |  |
| fasta-subsample | Extract a random selection of the sequences in a FASTA file. Can also subsample the sequences themselves. |  |  |
| fitevd | Fit an extreme value distribution to data. |  |  |
| gendb | Generate sequences from a Markov model. |  |  |
| getsize | Print statistics about sequences read from a FASTA file. |  |  |
| glam2mask | Mask glam2 motifs out of sequences, so that weaker motifs can be found. |  |  |
| gomo\_highlight | Identify GO terms which are implied by other GO terms, allowing the most specific GO terms to be highlighted in the conversion to html. |  |  |
| meme-io | Print summary of MEME HTML file to standard output as plain text. |  |  |
| meme-rename | Easily rename MEME Suite HTML files to unique names incorporating the path name (rather than "meme.html"). |  |  |
| pmb\_bf | Calculate the statistical power of phylogentic motif models. |  |  |
| psp-gen | Generate position-specific priors from positive (likely to contain a feature of interest) and negative (unlikely to contain a feature of interest) sequences for use as an additional input to MEME. |  |  |
| purge | Remove highly similar members of a set of sequences. |  |  |
| qvalue | Compute q-values from *p*-values. |  |  |
| reconcile-tree-alignment | Given a tree and an alignment, identify the intersection of the sets of sequence IDs and leaf labels. Trim the extra sequences and leaves and print the resulting alignment and tree. |  |  |
| reduce-alignment | Extract specified columns from a multiple alignment. |  |  |
| remove-alignment-gaps | Remove from an alignment all columns that correspond to a gap in a specified species. |  |  |
| shadow | Perform phylogenetic shadowing on a given DNA alignment, using a given tree. |  |  |

## File formats

- FASTA sequence files
  (with a sample)
- clustalw alignment files
- motif files
- background files
- TRANSFAC files
- GLAM2 alphabet files
- GLAM2 Dirichlet mixture files
- GOMO files
- GO DAG files

Accessing MEME Suite web applications from scripts

Installation instructions.

Release notes.

Visit the MEME Suite home page.


---

### Maintenance and development of the MEME Suite is funded by the National Center for Research Resources grant NIH/NCRR R01 RR021692. The MEME Suite web server is funded by the National Biomedical Computation Resource.

---

**Developed and maintained by:**

|  |  |  |  |
| --- | --- | --- | --- |
|  |  |  |  |

|  |  |  |
| --- | --- | --- |
|  |  |  |
